# *Sirt6* Regulates the Development of Medullary Thymic Epithelial Cells and Contributes to the Establishment of Central Immune Tolerance

**DOI:** 10.3389/fcell.2021.655552

**Published:** 2021-03-29

**Authors:** Qian Zhang, Zhanfeng Liang, Jiayu Zhang, Tong Lei, Xue Dong, Huiting Su, Yifang Chen, Zhaoqi Zhang, Liang Tan, Yong Zhao

**Affiliations:** ^1^State Key Laboratory of Membrane Biology, Institute of Zoology, Chinese Academy of Sciences, Beijing, China; ^2^University of Chinese Academy of Sciences, Beijing, China; ^3^Department of Central Laboratory and Institute of Clinical Molecular Biology, Peking University People’s Hospital, Beijing, China; ^4^Center of Organ Transplantation, Second Xiangya Hospital of Central South University, Changsha, China; ^5^Institute for Stem Cell and Regeneration, Chinese Academy of Sciences, Beijing, China

**Keywords:** autoimmune disease, immune tolerance, *Sirt6*, *Spib*, thymic epithelial cells (TECs), thymus

## Abstract

Although some advances have been made in understanding the molecular regulation of mTEC development, the role of epigenetic regulators in the development and maturation of mTEC is poorly understood. Here, using the TEC-specific *Sirt6* knockout mice, we found the deacetylase Sirtuin 6 (*Sirt6*) is essential for the development of functionally competent mTECs. First of all, TEC-specific *Sirt6* deletion dramatically reduces the mTEC compartment, which is caused by reduced DNA replication and subsequent impaired proliferation ability of *Sirt6*-deficient mTECs. Secondly, *Sirt6* deficiency specifically accelerates the differentiation of mTECs from CD80^–^Aire^–^ immature population to CD80^+^Aire^–^ intermediate mature population by promoting the expression of *Spib*. Finally, *Sirt6* ablation in TECs markedly interferes the proper expression of tissue-restricted antigens (TRAs) and impairs the development of thymocytes and nTreg cells. In addition, TEC conditional knockout of *Sirt6* results in severe autoimmune disease manifested by reduced body weight, the infiltration of lymphocytes and the presence of autoantibodies. Collectively, this study reveals that the expression of epigenetic regulator *Sirt6* in TECs is crucial for the development and differentiation of mTECs, which highlights the importance of Sirt6 in the establishment of central immune tolerance.

## Introduction

As a primary lymphoid organ, thymus plays an indispensable role in the establishment of central immune tolerance (Anderson and Takahama, 2012; Abramson and Anderson, 2017). Among the thymic stromal cells, thymic epithelial cells (TECs) provide a special microenvironment for the survival, development and maturation of thymocytes to the development of immunological competent T lymphocytes which can recognize foreign antigens rather than self-antigens (Sun et al., 2014; Abramson and Anderson, 2017; Nitta and Takayanagi, 2020). TECs include cortical thymic epithelial cells (cTECs) and medullary thymic epithelial cells (mTECs) (Alawam et al., 2020). cTECs are essential for positive selection of T lymphocytes (Takada et al., 2017). mTECs mediate negative selection by eliminating the autoreactive T lymphocytes and promoting the generation of nTreg cells, which are critical to build central immune tolerance and prevent the occurrence of autoimmune diseases (Akiyama et al., 2005; Anderson et al., 2007; Kadouri et al., 2020). The development of mTECs is mainly regulated by CD40, TNF receptor family (TNFRF) protein receptor activator of NF-κB (RANK), and lymphotoxin β receptor (LTβR) (Rossi et al., 2007; Zhu et al., 2007; Akiyama et al., 2008, 2012; Hikosaka et al., 2008; Seach et al., 2008). The cooperation of CD40 and RANK in mTECs is crucial for the establishment of self-tolerance (Akiyama et al., 2008; Hikosaka et al., 2008), while LTβR-mediated mTECs development is not involved in the establishment of self-tolerance (Cosway et al., 2017). Among the NF-κB signaling pathways, RelB subunit of the NF-κB complex (Burkly et al., 1995; Weih et al., 1995; Riemann et al., 2017; Jin and Zhu, 2018), NF-κB inducing kinase (NIK) (Kajiura et al., 2004), TNF receptor-activated factor 6 (TRAF6) (Akiyama et al., 2005), IκB-kinase (IKK) (Kinoshita et al., 2006), and NF-κB2 (Zhang et al., 2006; Zhu et al., 2006) have been widely recognized to regulate the development of mTEC and establish of self-tolerance. Compared with immature mTECs, functional mature mTECs act as the antigen present cell characterized by high expression of CD80, MHC class II (MHC II), and Aire (Kyewski and Klein, 2006; Klein et al., 2009; Anderson and Su, 2016). The expression of these molecules by mTECs is indispensable for thymocytes negative selection (Kadouri et al., 2020). Mature mTECs mediate the deletion of autoreactive T cells and promote the development of natural regulatory T cell (nTreg) by expressing tissue-restricted antigens (TRAs), which is essential for the establishment of central immune tolerance (Aschenbrenner et al., 2007; Cowan et al., 2013; Malhotra et al., 2016; Lebel et al., 2020). As so far, the molecular mechanisms regulating the development and differentiation of mTECs are still elusive. A recent report shows that SIRT1, a member of sirtuins family, could interact with Aire and induces its deacetylation, which is essential for Aire-driven TRAs expression and subsequent establishment of central immune tolerance (Chuprin et al., 2015). The epigenetic regulation of other Sirtuins family members in TEC development is not clear.

As a NAD^+^ dependent histone deacetylase, SIRT6 plays key roles in the regulation of metabolism, inflammation, longevity, genome stability and cancer (Kugel and Mostoslavsky, 2014; Chang et al., 2020). The phenotype of *Sirt6* knockout mice was early reported by Mostoslavsky et al. (2006). They found that *Sirt6* knockout mice died at about 4 weeks, the thymus of the mice diminished drastically and displayed a profound lymphopenia. The defects of lymphocytes in *Sirt6* knockout mice was non-cell-autonomous (Mostoslavsky et al., 2006), which implies the defect of thymus in *Sirt6* knockout mice may be caused by *Sirt6* ablation in thymic stromal cells. We therefore investigated the role of *Sirt6* in TECs using the TEC-specific *Sirt6* knockout mice. We found that *Sirt6* deficient in TECs caused severe thymic atrophy and that the proliferation, maturation and function of mTECs were drastically affected by *Sirt6* deletion. Impressively, TEC-specific *Sirt6* knockout mice spontaneously developed autoimmune disease. Our study unveils the indispensable role of *Sirt6* in the development and maturation of mTECs and in the establishment of central immune tolerance.

## Results

### TEC-Specific *Sirt6* Ablation Leads to Severe Thymic Atrophy

**FIGURE 1:**
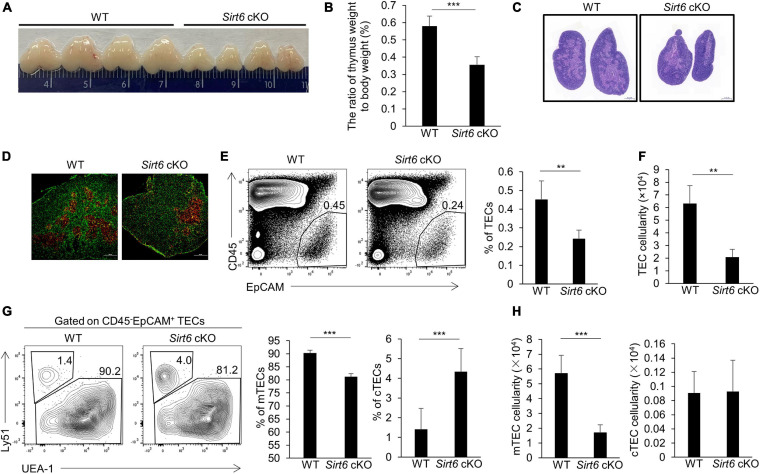
TEC-specific ablation of *Sirt6* causes thymus atrophy and reduces mTEC compartment. **(A)** Representatives of thymi isolated from 4-week-old *Sirt6* cKO mice and wild-type littermates. **(B)** Thymic weight normalized to body weight in 4-week-old wild-type and *Sirt6* cKO mice. **(C)** Hematoxylin and eosin (H&E) stained thymic sections of 4-week-old wild-type and *Sirt6* cKO mice. Scale bars: 1,000 μm. **(D)** Representative staining of frozen thymic sections of wild-type and *Sirt6* cKO mice. KRT8 (green) and KRT5 (red) highlights cortical regions and medullary regions, respectively. Scale bars: 500 μm. **(E)** Representative flow cytometric profiles and frequency of CD45^–^EpCAM^+^ TECs obtained from 4-week-old *Sirt6* cKO mice and littermates. **(F)** Total cell numbers of TECs of 4-week-old *Sirt6* cKO mice and littermates. **(G)** Representative flow cytometric profiles showing frequencies of mTECs (UEA-1^+^Ly51^–^) and cTECs (UEA-1^–^Ly51^+^) were first gated on TECs. **(H)** Total cell numbers of mTECs (left) and cTECs (right) of 4-week-old wild-type and *Sirt6* cKO mice. *N* = 4 per group. **p < 0.01 and ***p < 0.001 (Student’s *t*-test).

To investigate the role of *Sirt6* in TECs, we crossed *Foxn1*-cre mice (Soza-Ried et al., 2008; Liu et al., 2013) and *Sirt6*^flox/flox^ mice (Kim et al., 2010; Wang et al., 2016) to generate *Sirt6* conditional knock out mice (designated as *Sirt6* cKO mice henceforth) and we further confirmed *Sirt6* was indeed inactivated (Supplementary Figures 1A–C). First, we checked the effect of TEC-specific *Sirt6* knockout on the thymus of the 4-week-old mice. Compared with wild-type mice, the specific deletion of *Sirt6* in TECs led to severe thymic atrophy (Figures 1A,B). The morphological analysis showed that thymic medullary region decreased significantly while the cortical region had no obvious change after *Sirt6* ablation (Figures 1C,D).

As a longevity gene, whether *Sirt6* deletion leads to thymus senescence attracts our attention. Interestingly, although thymic atrophy still could be seen in 8-month-old *Sirt6* cKO mice, the extent of reduction in thymus size was smaller than that in 4-week-old *Sirt6* cKO mice (Supplementary Figure 2A). The rate of thymus weight loss (normalized for total body weight) and the rate of thymocytes number reduction did not increase in 8-month-old *Sirt6* cKO mice compared with wild-type control littermates (Supplementary Figure 2B). The morphology of thymus in 8-month-old *Sirt6* cKO mice showed no obvious signs of aging and the cortico-medullary junctions was integrated as assessed by H&E staining and Masson staining (Supplementary Figures 2C,D). Loss of naïve T cells in spleen is another indicator of age-related thymic degeneration (Aw and Palmer, 2012). However, from the 4 weeks to 8 months of age, the decline of naïve T cells in the spleen of *Sirt6* cKO mice did not worsen compared with control mice (Supplementary Figures 2E,F). Thus, we focused our studies on the role of *Sirt6* in the development and differentiation of TECs but not on the aging of TECs. Unless otherwise indicated, further experiments were carried out in 4-week-old *Sirt6* cKO mice and their littermate controls.

To assess the endogenous role of *Sirt6* in TECs, we checked the frequency and total number of TECs (CD45^–^EpCAM^+^) in *Sirt6* cKO mice and their littermate controls. Flow cytometry analysis revealed that *Sirt6* deficiency resulted in the reduction of TECs, both in proportion and in absolute cell number (Figures 1E,F). These results suggested the thymic hypoplasia in *Sirt6* cKO mice was caused by impaired TEC development. As we mentioned above, TECs are composed of cTECs and mTECs, we further scrutinized the effect of *Sirt6* ablation on cTECs and mTECs. We found the percentage and cell number of mTECs reduced remarkable and absolute number of cTECs was obviously unchanged although the proportion of cTECs was relatively increased in *Sirt6* cKO mice (Figures 1G,H). These results showed that *Sirt6* deficiency predominately influence the development of mTECs rather than cTECs.

### *Sirt6* Deficiency Impairs the Proliferation of mTECs by Reducing DNA Replication

**FIGURE 2:**
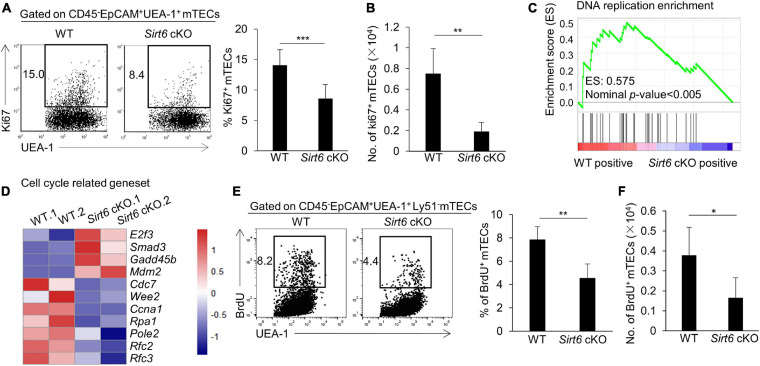
*Sirt6* deficiency impairs mTEC proliferation capacity by reducing DNA replication in cell cycle. **(A)** Flow cytometry plots and frequency for the staining of Ki67 in mTECs. **(B)** Cell numbers of Ki67^+^ mTECs of 4-week-old *Sirt6* cKO mice and littermates. **(C)** GSEA analysis reveals that DNA replication process had a less positive expression in *Sirt6* deficient mTECs compared with wild-type mTECs defined by the criterion of *p* < 0.005. **(D)** Heatmap of the significantly changed genes (*p* < 0.05) associated with cell cycle and proliferation. **(E)** BrdU staining was used for flow cytometry analysis of mTECs of 2-week-old *Sirt6* cKO mice and littermates 24 h after intraperitoneal injection of BrdU (1 mg per mice). **(F)** Cell numbers of BrdU^+^ mTECs of 2-week-old *Sirt6* cKO mice and littermates. *N* ≥ 4 per group. *p < 0.05, **p < 0.01, and ***p < 0.001 (Student’s *t*-test).

Due to the decreased thymus size and the cell number of mTECs in *Sirt6* cKO mice, we examined the proliferation and apoptosis of mTECs. The results showed that the percentage of Ki67-positive cells in total mTECs reduced by nearly half in *Sirt6* cKO mice (Figures 2A,B). Whereas, the expression of active caspase 3, an indicator of intrinsic apoptosis, did not increase in mTECs of *Sirt6* cKO mice compared with littermate controls (Supplementary Figure 3).

To better understand how *Sirt6* regulates cell proliferation, we performed RNA-sequencing analysis (RNA-seq) on mTECs (CD45^–^EpCAM^+^UEA-1^+^ly51^–^) sorted from 4-week-old wild-type or *Sirt6* cKO mice, with two parallel samples in each group (named WT.1 and WT.2, Sirt6 cKO. 1 and Sirt6 cKO. 2). Gene Set Enrichment Analysis (GSEA) showed that DNA replication process was significantly reduced in *Sirt6*-deficient mTECs (Figure 2C), indicating that *Sirt6* ablation may affect the cell cycle progression of mTECs. The expressions of many proliferation-related genes changed in *Sirt6* deficient mTECs (Figure 2D). The expressions of genes promoting cell cycle and cell proliferation such as *Cdc7, Ccna1, Rpa1, Pole2, Rfc2*, and *Rfc3* (Chen and Wold, 2014; Klajic et al., 2014; Tudzarova et al., 2016; Hu et al., 2020; Rogers et al., 2020) were down-regulated in *Sirt6* deficient mTECs compared with wild-type TECs, whereas genes inhibiting cell cycle and proliferation such as *E2f3*, *Smad3*, and *Gadd45b* (Salvador et al., 2013; Park et al., 2016; Clijsters et al., 2019) were significantly up-regulated in *Sirt6* deficient mTECs (Figure 2D). The modulated expressions of these genes would collectively contribute to the poor mTECs proliferation in *Sirt6* cKO mice. 5-bromo-2’-deoxyuridine (BrdU) incorporation assay was used to detect newly synthesized DNA in S phase. The portion and the absolute number of proliferating cell (BrdU-positive cells) decreased markedly in mTECs of 2-week-old *Sirt6* cKO mice in comparison with their littermate controls (Figures 2E,F). In contrast, *Sirt6* deficiency did not affect the proliferation of cTECs as showed by the similar BrdU incorporation (Supplementary Figure 4A).

### *Sirt6* Deficiency Promotes CD80 Expression on mTECs

**FIGURE 3:**
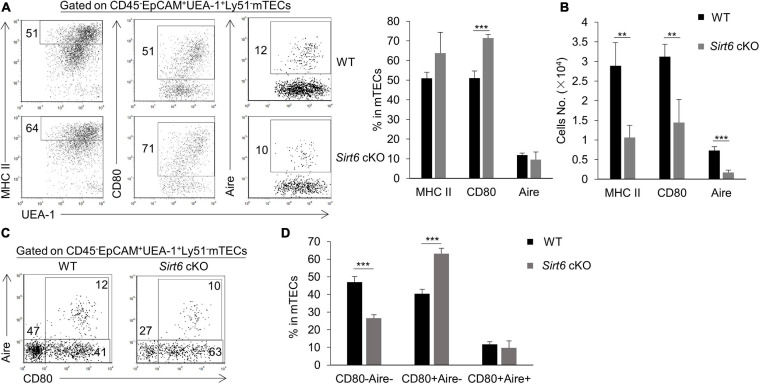
The proportion of CD80^+^ mTECs specifically increases in *Sirt6* cKO mice. **(A)** Representative flow cytometric profiles and frequencies of MHC II, CD80 and Aire expressed on mTECs of WT and *Sirt6* cKO mice. **(B)** Cell numbers of MHC II^high^, CD80^+^ and Aire^+^ mTECs of *Sirt6* cKO mice and littermates. **(C)** Flow cytometry plots showed the mature stage by detecting the expression of CD80 and Aire. **(D)** The ratio statistics of different stages in the maturation of mTECs have been shown. *N* ≥ 4 per group. **p < 0.01 and ***p < 0.001 (Student’s *t*-test).

We further investigated the effect of *Sirt6* deficiency on the differentiation and maturation of mTECs. Compared with immature mTECs, functional mature mTECs highly expressed CD80, MHC II and Aire, which were considered to play an critical role in negative selection (Kadouri et al., 2020). Flow cytometry analysis showed that the expression of MHC II and Aire on mTECs was similar between wild-type and *Sirt6* cKO mice, whereas the proportion of CD80^+^ mTECs increased significantly in *Sirt6* cKO mice (Figure 3A). Due to the overall reduction of mTECs, the absolute number of CD80^+^ mTECs, MHC II^high^ mTECs and Aire^+^ mTECs all decreased dramatically (Figure 3B). Specifically, Aire expressed on CD80^+^MHC II^high^ mTECs (mTEC^high^) and then regulated the expression of thousands of TRAs (Heino et al., 2000; Gray et al., 2007; Wells et al., 2020). *Sirt6* deletion accelerated the maturation of CD80^–^Aire^–^ mTECs to the differentiation of CD80^+^Aire^–^mTEC, but did not affect its further differentiation into CD80^+^Aire^+^ mTECs (Figures 3C,D). As shown by similar expression of MHC II and CD40, *Sirt6* did not affect the maturation of cTECs (Supplementary Figures 4B,C).

TNFR family members CD40, RANK, and LTβR play a critical role in promoting the development of mTECs (Rossi et al., 2007; Zhu et al., 2007; Akiyama et al., 2008, 2012; Hikosaka et al., 2008; Seach et al., 2008; Cosway et al., 2017). There was no significant difference in *Cd40* and *Rank* expression in mTECs between *Sirt6* cKO mice and wild-type mice, but the expression of *Ltbr* in *Sirt6* deficient mTECs decreased slightly (Supplementary Figure 5).

### *Sirt6* Regulates the Development and Maturation of mTECs Through SPIB-OPG Feedback Mechanism

**FIGURE 4:**
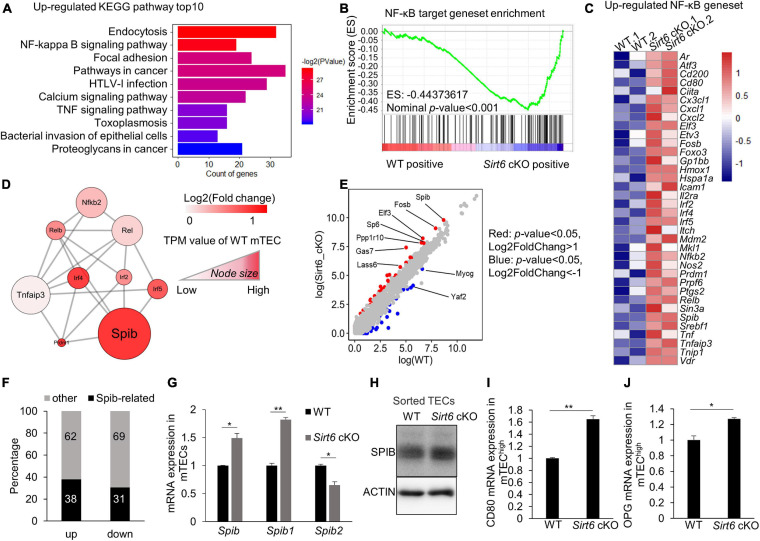
*Sirt6* deficiency leads to the activation of NF-κB pathway which in turn upregulates the expression of *Spib*. **(A)** Upregulated genes in *Sirt6* deficient mTECs were enriched in KEGG pathways, top10 pathways were ordered by *p*-value. All pathways were selected under the standard of *p* < 0.05. **(B)** GSEA analysis reveals that NF-κB target geneset had a more positive expression in *Sirt6* deficient mTECs defined by the criterion of *p* < 0.001. **(C)** The upregulated genes (*p* < 0.05) involved in NF-κB target geneset were performed by heatmap. **(D)** The molecular network between NF-κB and its downstream associated upregulated genes was constructed by STRING. All genes belong to transcription factors, color indicated the change of log2FoldChange and Node size indicated the TPM value of wild-type mTECs. **(E)** The scatter plot showed the difference of TPM values between wild-type and *Sirt6* deficient mTECs. The transcription factors with significant changes were color-coded in the plot, red indicated that genes under the criterion of *p* < 0.05 and log2foldchange > 1 and blue indicated that genes under the criterion of *p* < 0.05 and log2foldchange < -1. **(F)** The bar graph shows the changes of SPIB target genes in *Sirt6* deficient mTECs relative to wild-type mTECs. Black indicates the proportion of SPIB target genes, which was obtained from the results of ChIP-seq. **(G)** Expression of *Spib* and its two different promoters (*Spib1* and *Spib2*) in mTECs (CD45^–^EpCAM^+^UEA-1^+^Ly51^–^) sorted from wild-type or *Sirt6* cKO mice were measured by quantitative Real-Time PCR analysis. Data were normalized to *Hprt* mRNA levels. **(H)** Western blot result for SPIB expression in TECs (CD45^–^EpCAM^+^) sorted from wild-type or *Sirt6* cKO mice. **(I,J)** Quantitative Real-Time PCR analysis of *Opg* **(I)** and *Cd80* **(J)** mRNA expression in mTEC^high^ (CD45^–^EpCAM^+^UEA-1^+^CD80^+^MHC II^high^) sorted from wild-type and *Sirt6* cKO mice. Data were normalized to *Hprt* mRNA levels. *p < 0.05 and **p < 0.01 (Student’s *t*-test).

In order to figure out the regulation of mTEC maturation in molecular basis, we performed KEGG analysis for the RNA-seq results. We found that the endocytosis, NF-κB signaling pathway and focal adhesion were upregulated in *Sirt6*-deficient mTECs (Figure 4A). Among them, NF-κB signaling pathway has been reported to be essential in the development and maturation of mTECs (Burkly et al., 1995; Weih et al., 1995; Kajiura et al., 2004; Akiyama et al., 2005; Kinoshita et al., 2006; Zhang et al., 2006; Zhu et al., 2006; Riemann et al., 2017; Jin and Zhu, 2018). NF-κB target geneset was also enriched in *Sirt6* absent mTECs, indicating that NF-κB signaling pathway was indeed activated after *Sirt6* deletion (Figures 4B,C).

Next, we focused on the NF-κB signaling pathway to understand why *Sirt6* specifically restricts the development of CD80^+^ mTECs. We analyzed the network of NF-κB related transcription factor and found that *Spib*, as an important transcription factor related to the development and differentiation of mTEC (Akiyama et al., 2014), was notably increased in *Sirt6*-deficient mTECs (Figure 4D). Among the known transcription factors involved in the development and differentiation of mTEC, *Spib* showed the greatest difference between WT and *Sirt6* deficient mTECs (Supplementary Figure 6A). Furthermore, *Spib* was the highest expressed compared with other transcription factors that changed (*p* < 0.05) between *Sirt6* deficient mTECs and wild-type control (Figure 4E).

In order to verify the contribution of upregulated *Spib* in *Sirt6* deficient mTECs, we compared the genes directly regulated by *Spib* (Christie et al., 2015) and the genes changed in *Sirt6* deficient mTECs. Our RNA-seq results showed that 920 genes was down-regulated (*p* < 0.05), and 1,217 genes was up-regulated (*p* < 0.05) in the *Sirt6* deficient mTECs. Among the genes regulated by *Spib*, 461 genes were up-regulated (38% of 1,217 upregulated genes) and 281 genes were down-regulated (31% of 920 down-regulated genes) (Figure 4F). The genes directly regulated by *Spib* accounted for 35% of those with statistical difference between wild-type and *Sirt6* deficient mTECs.

Previous studies showed that *Spib1* rather than *Spib2* is highly expressed in mTECs (Akiyama et al., 2014). To verify the expression of *Spib* in mTECs, we isolated mTECs (CD45^–^EpCAM^+^UEA-1^+^ly51^–^) from wild-type or *Sirt6* cKO mice. Quantitative PCR analysis showed that *Spib1*, not *Spib2*, was upregulated after *Sirt6* deletion (Figure 4G). In addition, western blot result of sorted TECs (CD45^–^EpCAM^+^) confirmed that SPIB was indeed up-regulated after *Sirt6* conditional knockout (Figure 4H and Supplementary Figure 6B). It has been reported that *Spib* promotes *osteoprotegerin* (*Opg*) expression and participates in the mTEC developmental stage–specific negative feedback regulation (Akiyama et al., 2014). The development of mTECs was limited in *Sirt6* cKO neonates but not in E16.5 (Supplementary Figures 7A,B), which was consistent with the previous report that *Spib* regulated the development of mTECs in neonates rather than embryos (Akiyama et al., 2014). mTEC^hi^ population (CD45^–^EpCAM^+^UEA-1^+^CD80^+^MHC II^high^) were sorted from wild-type or *Sirt6* cKO mice (Supplementary Figure 6C), and the expression of *Cd80* and *Opg* were evaluated by quantitative PCR analysis. After *Sirt6* deletion, the expressions of *Cd80* and *Opg* were up-regulated (Figures 4I,J).

### The Development of Thymocytes Is Abnormal in *Sirt6* cKO Mice

**FIGURE 5:**
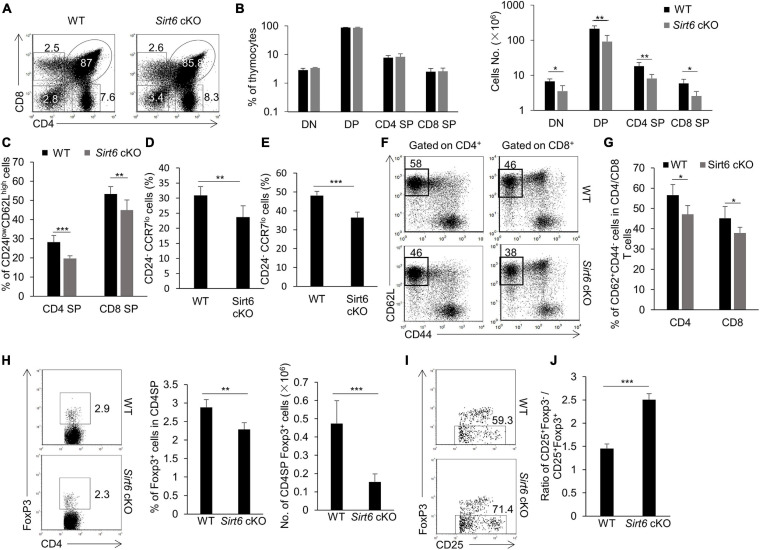
The development of thymocytes is impaired after *Sirt6* deletion in TECs. **(A)** Representative flow cytometry plots of CD4 and CD8 expressed on thymocytes derived from wild-type and *Sirt6* cKO mice. **(B)** Frequencies (left) and absolute cell numbers (right) of DN (CD4^–^CD8^–^), DP (CD4^+^CD8^+^), CD4SP (CD4^+^CD8^–^), and CD8 SP (CD4^–^CD8^+^) thymocytes of *Sirt6* cKO mice and littermate controls. **(C)** Frequencies of CD24^low^CD62L^high^TCRβ^+^ CD4SP and CD24^low^CD62L^high^TCRβ^+^ CD8SP mature thymocytes of wild-type and *Sirt6* cKO mice. **(D)** Frequencies of CD24^–^CCR7^lo^CD4^+^CD8^–^TCR^+^CD5^+^Foxp3^–^ thymocytes of wild-type and *Sirt6* cKO mice. **(E)** Frequencies of CD24^–^CCR7^lo^CD4^–^CD8^+^TCR^+^CD5^+^ thymocytes of wild-type and *Sirt6* cKO mice. **(F,G)** Representative flow cytometry plots **(F)** and frequency **(G)** of naïve (CD62L^+^CD44^–^) T cells in CD4^+^ or CD8^+^ splenocytes of wild-type and *Sirt6* cKO mice. **(H)** Flow cytometry plots (left) and frequency (middle) and absolute cell numbers (right) of Foxp3^+^nTreg of *Sirt6* cKO mice and littermate controls. **(I)** Flow cytometry plots show the maturation of nTreg from precursors (CD4^+^CD8^–^CD25^+^Foxp3^–^) to mature (CD4^+^CD8^–^CD25^+^Foxp3^+^) in wild-type and *Sirt6* cKO mice. **(J)** Ratio of precursor to mature nTreg between wild-type and *Sirt6* cKO mice. *N* ≥ 4 per group. *p < 0.05, **p < 0.01, and ***p < 0.001 (Student’s *t*-test).

Positive and negative selection T cells in thymus is mainly orchestrated by TECs (Anderson et al., 2000; Rodewald, 2008). We examined the development of thymocytes in *Sirt6* cKO mice to determine whether the impaired mTEC development affected its function in orchestrating thymocytes development. Although the proportion of DN, DP, CD4 SP, and CD8 SP was unaffected by *Sirt6* deletion, their cellularity decreased significantly in *Sirt6* cKO mice compared with wild-type control (Figures 5A,B). The further maturation of post-selected thymocytes downregulates the expression of CD24, CCR7 and upregulates the expression of CD62L (Zuklys et al., 2016; Liang et al., 2018). We found the frequency of CD24^low^CD62L^high^CD4^+^CD8^–^TCRβ^+^ and CD24^low^CD62L^high^CD4^–^CD8^+^TCRβ^+^ reduced in *Sirt6* cKO mice (Figure 5C and Supplementary Figure 8A). The percentage of CD24^–^CCR7^lo^CD4^+^CD8^–^TCRβ^+^CD5^+^Foxp3^–^ thymocytes and CD24^–^CCR7^lo^CD4^–^CD8^+^TCRβ^+^CD5^+^ thymocytes decreased in *Sirt6* cKO mice (Figures 5D,E and Supplementary Figure 8B), implying the further maturation of thymocytes was restrained by dysfunctional mTECs in *Sirt6* cKO mice. The number of CD4^+^ T cells or CD8^+^ T cells and the portion of naïve CD4^+^ T cells or naïve CD8^+^ T cells reduced in spleen, indicating that T cells output from thymus decreased after *Sirt6* deletion in TECs (Figures 5F,G and Supplementary Figure 8C).

In addition to the elimination of self-reactive T cells, mTECs play an important role in promoting the diversion of nTreg cells lineage (Cowan et al., 2013; Kadouri et al., 2020). The results showed that the percentage and the cell number of mature nTreg cells (Foxp3^+^CD4 SP) decreased obviously in *Sirt6* cKO mice compared to their littermate controls (Figure 5H). By detecting the maturation process of nTreg cells (Lio and Hsieh, 2008), we found the ratio of immature nTreg cells (Foxp3^–^CD25^+^CD4 SP) to mature nTreg cells (Foxp3^+^CD25^+^CD4 SP) increased notably in *Sirt6* cKO mice, implying that the maturation of nTreg cells was blocked during the differentiation of Foxp3^–^CD25^+^CD4 SP to Foxp3^+^CD25^+^CD4 SP (Figures 5I,J and Supplementary Figure 8D).

### Central Immune Tolerance Is Disrupted in the *Sirt6* cKO Mice

**FIGURE 6:**
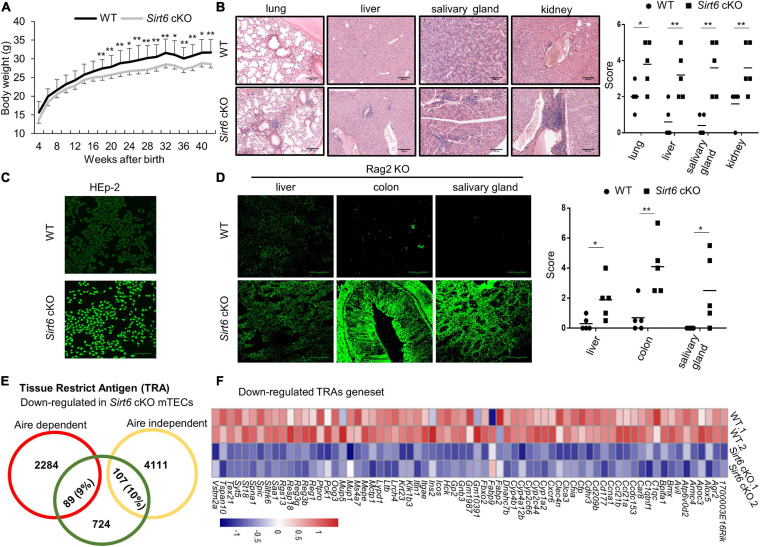
*Sirt6* cKO mice spontaneously develop severe autoimmune disorder. **(A)** Curve of body weight with age of wild-type and *Sirt6* cKO mice. **(B)** Hematoxylin and eosin (H&E) stained paraffin embedded sections of lung, liver, salivary gland, and kidney of 8-month-old wild-type and *Sirt6* cKO mice. Infiltration scores and means are indicated. Scale bars: 200 μm. **(C)** Antinuclear antibodies from the serum of 8-month-old wild-type and *Sirt6* cKO mice combined with HEp-2 cell line then detected by anti-mice IgG-AF488 antibody. Scale bars: 100 μm. **(D)** Tissue sections (liver, colon, and salivary gland) of Rag2 knockout mice were incubated with the serum of 8-month-old wild-type and *Sirt6* cKO mice to detect autoantibodies. Scores and means are indicated. Scale bars: 100 μm. **(E)** The downregulated of Aire-dependent and Aire-independent TRA genes in the comparison of wild-type and *Sirt6* deficient mTEC were calculated in venn diagram. Aire-dependent genes were circled in red and Aire-independent genes were circled in yellow. **(F)** Heatmap of the downregulated TRAs genes (*p* < 0.05) in *Sirt6* deleted mTEC. *N* ≥ 5 per group. *p < 0.05 and **p < 0.01 (Student’s *t*-test).

Due to the developmental and functional defects of mTECs in *Sirt6* cKO mice, we next determined whether such defects affected the establishment of central immune tolerance. We compared the body weight of *Sirt6* cKO mice with their age-matched littermate controls and found the body weight of *Sirt6* cKO mice was lower obviously than their age-matched littermate controls 20 weeks after birth (Figure 6A). Lymphocytic infiltrates in multiple organs is an important index to judge the occurrence of autoimmune disease (Chuprin et al., 2015). H&E staining results showed that many organs in the 8-month-old *Sirt6* cKO mice had more severe lymphocytes infiltration than wild-type control mice, including the salivary gland, kidney, lung, and liver (Figure 6B). We further examined the presence of autoantibodies in serum of 8-month-old *Sirt6* cKO. Indeed, there were high levels of antinuclear antibodies in the serum of *Sirt6* cKO mice (Figure 6C). What’s more, compared with wild-type control mice, *Sirt6* cKO mice had more autoantibodies against many organs of Rag2 KO mice (Shinkai et al., 1992), such as liver, colon, and salivary gland (Figure 6D).

The expression of TRAs on the mature mTECs is essential for eliminating auto-reactive T cells and promoting the development of nTreg cells, which is essential for the establishment of central immune tolerance (Klein et al., 2000). We analyzed the expression of Aire-dependent and Aire-independent TRAs (Sansom et al., 2014; Rodrigues et al., 2017) and found that the expression of both Aire-dependent and Aire-independent TRAs was affected in the mTECs after *Sirt6* deletion (Figure 6E). Compared with *Sirt1* which only specifically regulates Aire-dependent TRAs (Chuprin et al., 2015), *Sirt6* affect a wider range of TRAs expression. Most of the down-regulated TRAs in *Sirt6* deficient mTECs were shown in Figure 6F. The decreased TRAs expression indicated that the function of mTECs in establishing central immune tolerance was impaired after *Sirt6* deletion.

## Discussion

We define *Sirt6* as an important regulator in mTEC development. As *Sirt6* ablation inhibits the proliferation of mTECs, the percentage and absolute number of mTECs decrease significantly. These results suggest that, in contrast to *Sirt1*, *Sirt6* is involved in the regulation of mTEC development. Previous reports have shown that *Sirt6* deficiency has linked to many diseases, such as cancer, neurodegeneration and aging (Khan et al., 2018). Because *Sirt6* knockout mice died several weeks after birth (Mostoslavsky et al., 2006), the specific function of *Sirt6* in different organs are unclear. Age-related thymic involution occurs at the early stage of organisms (Taub and Longo, 2005; Chinn et al., 2012) and TEC-intrinsic molecules are considered to be an important and sufficient to initiate thymus involution (Lynch et al., 2009; Rezzani et al., 2014; Cheng and Anderson, 2018; Wang et al., 2019; Baran-Gale et al., 2020). Although a recent study showed that *Sirt6* deficiency in cynomolgus monkey causes developmental retardation, the main effect of *Sirt6* deficiency in rodents was showed to be associated with accelerated aging (Mostoslavsky et al., 2006; Kawahara et al., 2009; Kanfi et al., 2012; Zhang et al., 2018). Compared with thymus weight loss and naïve T cell levels in spleens of wild-type and *Sirt6* cKO mice during 4 weeks to 8 months after birth, *Sirt6* cKO mice do not show any detectable increased thymus weight loss and the reduced naïve T cells in spleen. These results suggest that *Sirt6* ablation in TECs do not accelerate thymic involution.

On the other hand, we have found that 8-month-old *Sirt6* cKO mice, rather than age-matched wild-type mice, have substantial lymphocytic infiltrates in multiple organs and obvious autoantibodies in the serum, indicating that *Sirt6* deficient in mTECs leads to autoimmune disorders. These pathological changes can be supported by the decrease of Aire-dependent and independent TRA expressions and the blockage of nTreg cell maturation in the thymus of *Sirt6* cKO mice. The impaired TRA expression and the poor nTreg cell maturation in *Sirt6* cKO mice may lead to the failure of establishing central immune tolerance, which subsequently contribute to the development of autoimmune disease in *Sirt6* cKO mice. On the other hand, the frequency of Helios expressing thymocytes during T cell maturation (Daley et al., 2013; Zuklys et al., 2016) in *Sirt6* cKO mice did not change compared with those in WT mice, indicating that the negative selection of these cells in Sirt6 cKO mice seems not remarkably affected (Data not shown). However, the development of CD24^+^CCR7^+^ to CD24^–^CCR7^lo^ CD4SP and CD8 SP cell in Sirt6 cKO mice was blocked, and the frequency of TCRβ^high^CD24^low^CD62L^high^ CD4 SP and TCRβ^high^CD24^low^CD62L^high^ CD8 SP in Sirt6 cKO mice were decreased, implying the further maturation of T cells may be restrained by dysfunctional mTECs in *Sirt6* cKO mice. It is reported that chemokines CCL19 and CCL21, as functional ligands of CCR7, play an important role in the accumulation of positively selected thymocytes in thymic medulla, which is crucial for the elimination of auto-reactive T cells (Ueno et al., 2002, 2004; Anderson and Takahama, 2012; Kozai et al., 2017). RNA-seq data showed that the expression of Ccl19 and Ccl21a in Sirt6 deficient mTECs decreased significantly, which indicated that the ability of mTEC to eliminate self-reactive T cells might be impaired. Previous reports show that different GFP levels in Rag2p GFP transgenic mice can be used to evaluate thymocyte medullary dwell time, “thymic age” of thymocytes and identify recent thymic emigrants directly (Yu et al., 1999; Boursalian et al., 2004; McCaughtry et al., 2007; Hauri-Hohl et al., 2014; Cowan et al., 2016; White et al., 2017). The medullary residency time is closely related to the establishment of central immune tolerance (McCaughtry et al., 2007). By using the OT2/RIP-OVA system, self-reactivity and TCR affinity for self-antigen are proved to be related to the negative selection and the development of nTreg cells (Lee et al., 2012; Wyss et al., 2016; Santamaria et al., 2021). Thus, we should employ these mouse models to directly and systemically address whether thymic negative selection and Treg development are impaired by *Sirt6* deficiency in TECs in the future.

The results showed that the proportion and the cell number of Ki67^+^ mTECs and BrdU^+^ mTECs decreased after *Sirt6* deletion. Together with the analysis of RNA-seq assays, our data indicate that the reduced proliferation ability of *Sirt6*-deficient mTECs is likely due to the decrease of DNA replication. The differentiation from CD80^–^ mTECs to CD80^+^ mTECs has been accelerated specifically in *Sirt6* cKO mice, implying the differentiation of mTECs is affected in the absence of *Sirt6*. SIRT6 is originally thought to be a mono-ADP-ribosyltransferase, which has later been found to be involved in DNA repair (Liszt et al., 2005; Mao et al., 2011). SIRT6, on the other hand, is widely known as a deacetylase (Michishita et al., 2008; Kawahara et al., 2009; Michishita et al., 2009; Yang et al., 2009; Zhong et al., 2010; Tasselli et al., 2016). In sorted mTECs, acetylation do not increase after *Sirt6* ablation, indicating that SIRT6 may not act as a key deacetylase in the development of mTEC. This observation is supported by the studies showing that deacetylase activity of SIRT6 is 1,000 times slower than other sirtuin family members (Pan et al., 2011). Our RNA-seq analysis data show that NF-κB signaling pathway is remarkably up-regulated in the *Sirt6*-deficient mTECs, which is nicely in line with the observation showing that *Sirt6* deletion up-regulated the expression NF-κB target genes (Kawahara et al., 2009). It is well-known that the development and differentiation of mTEC depends on NF-κB signaling pathway (Burkly et al., 1995; Weih et al., 1995; Kajiura et al., 2004; Akiyama et al., 2005; Kinoshita et al., 2006; Zhang et al., 2006; Zhu et al., 2006; Riemann et al., 2017; Jin and Zhu, 2018). The enhanced NF-κB signal pathway up-regulated the expression of *Spib* in mTECs after *Sirt6* deletion as indicated by RNA-seq, real-time PCR and western blot assays, as previously reported (Akiyama et al., 2014). It is nicely demonstrated by Akiyama et al. that *Spib* remarkably limits the development and maturation of mTECs but promotes CD80 expression in mature mTECs (Akiyama et al., 2014). Importantly, RANKL stimulation consecutively induces *Spib* expression in TECs and *Spib* facilitates expression of OPG protein, which competitively inhibits RANKL–RANK interactions as a decoy receptor of RANKL, in mTECs by maintenance of the hypomethylated states in *Opg* (Akiyama et al., 2014; Tsukasaki et al., 2020). Thus, SPIB–mediated negative feedback regulation of RANKL signaling limits mTEC development in neonates but not in embryos by forming RANKL-NF-κB-SPIB-OPG regulating loop in mTECs (Akiyama et al., 2014). Our results suggest that *Sirt6* negatively regulates the expression of NF-κB-SPIB-OPG pathway, which is crucial for the postnatal development and maturation of mTECs. However, it should be noted that mTECs are heterogenous and some atypical types of terminally differentiated mTECs exist in the thymus (Bornstein et al., 2018; Miller et al., 2018; Park et al., 2020). A newly defined IL25^+^ thymic tuft cells are regulated by *Pou2f3* and affect the development of thymus-resident type-2 innate lymphoid cells and thymic invariant natural killer T cells (Bornstein et al., 2018; Miller et al., 2018; Lucas et al., 2020). The latest research shows that there are two distinct groups in human TECs, TEC(myo)s and TEC(neuro)s (Park et al., 2020). Considering the diversity and heterogeneity of TECs, we may speculate another possibility that the increased *Spib* expression in Sirt6 cKO mTECs may be caused by the enhanced *Spib* expression in some unidentified mTEC subpopulation rather than that the expression level of *Spib* was simply increased in whole mTECs. Unfortunately, our present results could not exclude this possibility and more detailed studies on *Sirt6*-mediated regulation in the development of mTEC subpopulations should be performed in the future.

In summary, our study reveals that *Sirt6* is involved in the regulation of the development, maturation and function of mTECs and is critical for establishment of central immune tolerance. These results support the non-redundant role of different epigenetic molecules in maintaining the functional integrity of mTEC and preventing autoimmune disorders.

## Materials and Methods

### Mice

*Sirt6*^loxp/loxp^ mice (Kim et al., 2010; Wang et al., 2016) were crossed with *Foxn1*-Cre mice (Soza-Ried et al., 2008; Liu et al., 2013) to generate *Foxn1*-Cre *Sirt6*^loxp/loxp^ mice. Littermates or age-matched wild-type mice (*Foxn1*-Cre negative) were used as controls. We obtained *Sirt6*^loxp/loxp^ mice from Dr. Zhenyu Ju of Hangzhou Normal University, Hangzhou, China, and obtained *Foxn1-*Cre mice from Dr. Yu Zhang of Peking University Health Science Center, Beijing, China. Rag2 KO mice (Shinkai et al., 1992) was purchased from HFK Bioscience, Beijing, China. All mice were maintained under specific pathogen–free conditions and treated in accordance with Animal Experiments Guidelines of the animal Ethics Committee of Institute of Zoology, Beijing, China.

### Thymic Stromal Cell Isolation

Thymic stromal cells was isolated from whole thymus by using the previous thymic stromal cell separation method (Sun et al., 2013; Liang et al., 2018). In brief, fresh thymus tissue was cut into pieces and suspended the thymus fragments in DMEM (Hyclone Laboratories, SH30022.01B) medium with 2% fetal bovine serum (FBS; Gibco, 16000-044). Most thymocytes in the supernatant were removed, and the remaining thymus fragments were incubated at 37°C for 15 min in 2ml solution of 1 mg/ml collagenase/dispase (Sigma-Aldrich, 11097113001) with 20 U/ml DNAse I (Sigma-Aldrich, D5025). Repeat the above digestion three times, gently shaking to make digestion more thorough. The digested cell suspension was terminated with PBS containing 1% FBS and 5 mM EDTA until all fragments disappeared. After centrifugation, cells were suspended in DMEM (containing 2% FBS). Cell suspension was gently blown and filtered with a 200 mesh filter to remove clumps and form a single cell suspension.

### Flow Cytometric Analysis and Antibodies

Fc-receptor was blocked by 2.4G2 before staining with indicated antibodies. All type of cell were stained with the appropriate fluorophore-labeled antibodies at 4°C for 30 min. For intracellular staining, the fixation buffer (eBioscience, 00-5123-43 and 00-5223-56) and permeabilization buffer (eBioscience, 00-8333-56) was used according to the supplier’s protocol. The flow cytometry was performed with Gallios Flow Cytometer (BeckMan Coulter, United States) or BD LSRFortessa X-20 Flow Cytometer (BD Biosciences, United States).

The Fluorescein labeled Ulex Europaeus Agglutinin I (UEA I) (Vector Laboratories, FL-1061) was obtained from Vector Laboratories. The CD45-PerCP/Cy5.5 (Biolegend, clone 30-F11, 103132), CD326-PE/Cy7 (Biolegend, clone G8.8, 188216), CD326-FITC (Biolegend, clone G8.8, 118207), Ly51-AF647 (Biolegend, clone 6C3, 108312), CD40-PE (Biolegend, clone 3/23, 124610), I-A/I-E-Brilliant Violet 421 (Biolegend, clone M5/114.15.2, 107632), CD80-PE (Biolegend, clone 16-10A1, 104708), CD80-BV650 (Biolegend, clone 16-10A1, 104732), CD4-FITC (Biolegend, clone GK1.5, 100405), CD4-PE (Biolegend, clone GK1.5, 100408), CD4-PE/Cy5 (Biolegend, clone GK1.5, 100410), CD4-APC (Biolegend, clone GK1.5, 100412), CD4-APC/Cy7 (Biolegend, clone GK1.5, 100412), CD8a-PE (Biolegend, clone 53-6.7, 100725), CD8a-PE/Cy5 (Biolegend, clone 53-6.7, 100710), CD8a-Brilliant Violet 421 (Biolegend, clone 53-6.7, 100738), TCR-β-PE-Cy7 (Biolegend, clone H57-597, 109222), CD24-FITC (Biolegend, clone M1/69, 101805), CD24-PE/Cy5 (Biolegend, clone M1/69, 101812), CD44-FITC (Biolegend, clone IM7, 103006), CCR7-PE (Biolegend, clone 4B12, 120106), CD5-APC (Biolegend, clone 53-7.3, 100626) were purchased from Biolegend. The Aire (eBioscience, clone 5H12, 50-5934-80), Fixable Viability Dye eFluor^TM^ 506 (eBioscience, 65-0866-18), CD62L-PE (eBioscience, clone MEL-14, 12-0621-82), CD25-PE-Cy5 (eBioscience, clone PC61.5, 12-0251-82), Foxp3-FITC (eBioscience, clone FJK-16s, 11-5773-82), were purchased from eBioscience. CD45-BUV395 (BD Biosciences, clone 30-F11, 564279), Ly51-BV786 (BD Biosciences, clone BP-1, 740882), PE Mice Anti-Ki-67 Set (BD Biosciences, 556027), APC BrdU Flow Kit (BD Biosciences, 552598), and PE Active Caspase-3 Apoptosis Kit (BD Biosciences, 550914) were purchased from BD Biosciences.

### BrdU Incorporation Assays

For Brdu incorporation, 2-week-old *Sirt6* cKO and littermate control mice were intraperitoneally injected with BrdU (BD Biosciences), 1 mg per mice. Twenty-four hours after injection, thymic stromal cells were isolated according to the above-mentioned method before flow cytometric analysis. After surface antigens staining, according to the supplier’s protocol (BD Pharmingen^TM^ BrdU Flow Kits Instruction Manual), BrdU was detected by using APC-BrdU Flow Kit (BD Biosciences, 552598) and was analyzed by flow cytometry.

### Immunofluorescence Staining

Tissues were embedded in optimum cutting temperature compound (Sakura, 4583) and frozen in liquid nitrogen. Sections (6 μm in thickness) and/or cells were fixed for 20 min with 4% polyoxymethylene (Solarbio, P1110) and blocked in PSB containing 1% BSA. Then, sections and/or cells were incubated with primary and secondary antibodies for 1 h at room temperature. Samples were stained with DAPI (1:1,000) after secondary staining. The following antibodies were used for staining: rabbit anti-KRT5 (Covance, PRB-160P; clone AF 138) diluted by 1:400 and rat anti-KRT8 (DSHB, AB 531826; Troma-I) diluted by 1:200. Sera of 8-month-old wild-type and *Sirt6* cKO mice (diluted by 1:30) were used as primary antibodies for the detection of the autoantibodies. The secondary antibodies were used for staining: Alexa Fluor 594-conjugated donkey anti-rabbit IgG (H + L) (Jackson ImmunoResearch Laboratories, 711-586-152) diluted by 1:400, Alexa Fluor 488-conjugated donkey anti-rat IgG (H + L) (Jackson ImmunoResearch Laboratories, 712-546-150) diluted by 1:400, and Alexa Fluor 488-conjugated donkey anti-mice IgG (H + L) antibodies (Jackson ImmunoResearch Laboratories, 715-546-150) diluted by 1:300. All antibodies were diluted in 0.5% BSA in PBS. Laser scanning confocal microscope (Zeiss LSM710, Oberkochen, Germany) were used to acquire images.

### RNA-Seq Data Processing

As mentioned above, thymic stromal cells were isolated from 4-week-old wild-type and *Sirt6* cKO mice, with two independent parallel samples in each group. The next RNA sequencing step of sorted mTECs (CD45^–^EPCAM^+^UEA-1^+^Ly51^–^) was performed according to the previous method (Liang et al., 2020).

Low quality reads (*Q* < 20) were assessment by FastQC and the adaptor sequence was filtered by Trimgalore. Processed reads were aligned to mice genome (mm10) via HISAT2 (Pertea et al., 2016). StringTie was applied to assemble and quantify the transcripts in each sample to obtain the number of exon, transcription initiation/stop site, count and TPM (Transcripts Per Kilobase of exon model per Million mapped reads) values. The identification of differential expression genes (DEGs) was performed by using the DEseq2 (Love et al., 2014) R-packages with count value. The threshold is under the condition of adjusted *p* < 0.05 and | Foldchange| > 2. The normalized gene expression only retained the gene with TPM > 0.1 in all samples.

### KEGG Pathway Enrichment Analysis

The KEGG pathway analysis of the differentially expressed genes were performed by KOBAS 3.0 on the web^1^ (Ai and Kong, 2018). In addition, GSEA was carried out by searching KEGG Database (Subramanian et al., 2007). All analyses were selected with *p* < 0.05 as the cut-off criterion.

### Network Creation and Customization

Functional protein association network was created by STRING (Szklarczyk et al., 2015) (Available online: https://string-db.org/) which was constructed by the transcriptional factors obtain by website (Available online: http://bioinfo.life.hust.edu.cn/AnimalTFDB/). Networks were all visualized by Cytoscape (Demchak et al., 2014).

### Culture of TECs

Thymi of wild-type and *Sirt6* cKO neonatal mice were cut into small pieces and suspended with DMEM containing 2% FBS. After the small pieces settled at the bottom of the tube, the cell suspension was discarded. The remaining small pieces were suspended in TyEpiCM (ScienCell Research Laboratories, Catalog #3911) and incubator at 37°C with 5% CO_2_ for 7 days, exchanging the medium every other day.

### Western Blot Assay

Cultured wild-type and *Sirt6* cKO TECs were used to detect SIRT6 expression. TECs (CD45^–^EPCAM^+^) isolated from wild-type and *Sirt6* cKO mice were used to detect the expression of SPIB. After being washed with cold PBS, TEC were lysed in RIPA buffer (140 mM NaCl, 10 mM Tris-Cl (pH 8.0), 1 mM EDTA, 0.5 mM EGTA, 1% Triton X-100, 0.1% sodium deoxycholate and 0.1% SDS) complemented with a proteinase inhibitor cocktail (Sigma-Aldrich, P8340). Protein concentration was detected with Bradford assay. Proteins were analyzed by 10% SDS-PAGE and transferred onto PVDF membranes (Merck Millipore, IPFL00010). Each PVDF membrane was blocked with 5% non-fat dried milk (OXOID, LP0031) for 60–90 min at room temperature and incubated with each primary antibodies overnight on the shaking table at 4°C. After cleaning PVDF membrane with TBST solution for four times, the corresponding secondary antibodies were added for 45–60 min at room temperature. Protein bands were detected by chemiluminescence (Merck Millipore, WBKLS0500). ACTIN is used as internal reference for protein standardization. The primary antibodies used for western blot are as follows: Anti-SIRT6 (Cell Signaling Technology, 12486) diluted by 1:1,000; anti-SPIB (Cell Signaling Technology, 14337S) diluted by 1:1,000; anti-ACTIN (Sigma-Aldrich, A5441) diluted by 1:20,000.

### Quantitative RT-PCR

Thymic stromal cell was isolated from 4-week-old wild-type or *Sirt6* cKO mice and then sorted with MoFlo XDP cell sorter (Beckman Coulter, Brea, CA, United States), characterized as TEC (CD45^–^EPCAM^+^), mTEC (CD45^–^EPCAM^+^UEA-1^+^Ly51^–^), cTEC (CD45^–^EPCAM^+^UEA-1^–^Ly51^+^), mTEC^low^ (CD45^–^EPCAM^+^UEA-1^+^CD80^–^MHCII^low^) and mTEC^high^ (CD45^–^EPCAM^+^UEA-1^+^CD80^+^MHCII^high^). Total RNA was isolated with MicroElute Total RNA Kits (Omega Bio-tek, R6831) and reverse transcription was performed with Super-Script III Reverse Transcription (Invitrogen, 18080–093) according to manufacturer’s instructions.

Real-time PCR was performed with multiple kits (SYBR Premix Ex Taq, Takara Bio, DRR041A) on CFX96 (Bio-Rad Laboratories, Hercules, CA, United States). All primers used in this article were listed in Table 1.

**TABLE 1:** The list of primers used in qPCR assays.

Gene	Forward	Reverse
*Hprt*	TGAAGAGCTACTGTAATGATCA GTCAA	AGCAAGCTTGCAACCTTAACCA
*Sirt6*	GGCAGTCATTGTCTCCACCA	TCTCGAAGGTGGTGTCAAAC
*Cd40*	CTGTGAGGATAAGAACTTGGAGG	AGAGAAACACCCCGAAAATGG
*Rank*	TCTCAGATGTCTTTTCGTCCAC	CTCAGTGTCATGGAAGAGCTG
*Ltbr*	CAACCCCATACCAGATGTGAG	GAAGAGCAGAAAGAGGACCAG
*Spib*	CTGCAAGCCCTTCAGTTACC	AAAGGCAGCAGTAGCAGGAT
*Spib1*	CTCTGAACCACCATGCTTGCT	TCCTTCTGGGTACAAACAGCTTAA
*Spib2*	AGGGCGGCCCTGACAT	TCCTTCTGGGTACAAACAGCTTAA
*Cd80*	GCTGATTCGTCTTTCACAAGTG	GCCAGTAGATTCGGTCTTCAG
*Opg*	GGGCGTTACCTGGAGATCG	GAGAAGAACCCATCTGGACATTT
*Fabp2*	GCTGATTGCTGTCCGAGAGGTT	AGCCTGGCATTAGCATGATGGA
*Ins2*	GGAGGCTCTCTACCTGGTGTGT	TCTACAATGCCACGCTTCTGCT
*Mup1*	TGGCCGAGAACCAGATTTGAGT	GAGGCAGCGATCTGTAGTGTGA
*Resp18*	TCGAGGAACCGTGAGTTTGG	AGCTGTTCCGATCCCACTTG
*Apoc3*	CACAGAAGGCTTGGGACTCA	GACCGTCTTGGAGGCTTGTT
*Cd177*	GAGCTACCTACACCCACAGTTC	CCCTGCACCTTGAGATTGGT
*Pck1*	CAGCTGCTGCAGAACACAAG	CCGGAACCAGTTGACATGGA
*Fabp9*	TCGGTTGTGAATGCCTGGTCTG	TGCACTTCCTGCTTGGATGTCC

### Statistical Analysis

All data are presented as the means ± SD. The statistical significance of differences between two experimental groups was tested by Student’s *t*-test. A *p*-value < 0.05 was considered statistically significant.

## Data Availability Statement

The data presented in the study are deposited in the NCBI Gene Expression Omnibus public repository, accession number GSE166840.

## Ethics Statement

The animal study was reviewed and approved by the Animal Ethics Committee of Institute of Zoology, Beijing, China.

## Author Contributions

QZ, ZL, and TL designed and carried out the experiments, analyzed data, and wrote the manuscript. JZ performed the bioinformatics analyses and wrote the manuscript. XD, HS, and YC performed the experiments. ZZ performed the fluorescence staining. LT genotyped the genetically modified mice. YZ designed the experiments, analyzed the data, wrote the manuscript, and provided overall supervision. All authors contributed to the article and approved the submitted version.

## Conflict of Interest

The authors declare that the research was conducted in the absence of any commercial or financial relationships that could be construed as a potential conflict of interest.

## Footnotes

1 http://kobas.cbi.pku.edu.cn/kobas3/

## Supplementary Material

Supplementary Figure 1 SIRT6 was successfully knockout in TECs. **(A)** Identification method of WT (*Foxn1*.cre^–^) and *Sirt6* cKO (*Foxn1*.cre^+^, *Sirt6*^loxp/loxp^) mice. **(B)** Western blot result for *Foxn1*.Cre-mediated recombination efficacy of *Sirt6*^loxp/loxp^ between wild-type TECs and *Sirt6* cKO TECs cultured *in vitro*. **(C)** Quantitative Real-Time PCR analysis of *Sirt6* mRNA expression in mTEC (CD45^–^EpCAM^+^UEA-1^+^Ly51^–^) and cTEC (CD45^–^EpCAM^+^UEA-1^–^Ly51^+^) sorted from wild-type and *Sirt6* cKO mice. Data were normalized to *Hprt* mRNA levels.

Supplementary Figure 2 Aging-like phenotype did not observed in *Sirt6* cKO mice. **(A)** Representative figure of thymi isolated from 8-month-old WT and *Sirt6* cKO mice. **(B)** Thymic weight normalized to body weight (left) and the thymocytes cellularity (right) in 4-week-old WT and *Sirt6* cKO mice and 8-month-old WT and *Sirt6* cKO mice. WT (4-week-old: *n* = 4; 8-month old: *n* = 5) and *Sirt6* cKO mice (4-week-old: *n* = 4; 8-month-old: *n* = 9). **(C,D)** Hematoxylin and eosin (H&E) staining **(C)** and Masson’s Trichrome staining **(D)** stained sections of thymi from 8-month-old WT and *Sirt6* cKO mice. Scale bars: 500 μm. **(E,F)** Representative flow cytometric profiles **(E)** and frequency **(F)** of naïve T cells (CD62L^+^CD44^–^) in CD4^+^ (left) or CD8^+^ (right) splenocytes from 8-month-old or 4-week-old WT and *Sirt6* cKO mice (4-week-old: *n* = 14 per group; 8-month-old: *n* = 10 per group).

Supplementary Figure 3 Decreased mTECs in *Sirt6* cKO mice is not caused by apoptosis. Flow cytometry plots and frequency for the staining of Active Caspase3 in WT and *Sirt6* deficient mTECs. *N* = 4 per group. ^∗∗^*p* < 0.01 (Student’s *t*-test).

Supplementary Figure 4 The maturation and proliferation of cTECs were not influenced in *Sirt6* cKO mice. **(A)** Flow cytometry plots and frequency for BrdU^+^ cTECs from 2-week-old *Sirt6* cKO mice and littermates. **(B)** Representative flow cytometry plots and frequencies of CD40 (upper) and MHC II (lower) expressed on cTECs. **(C)** Cell numbers of CD40^+^ cTECs (upper) and MHC II^high^ cTECs (lower) of 4-week-old *Sirt6* cKO mice and littermates. *N* ≥ 4 per group. ^∗^*p* < 0.05 (Student’s *t*-test).

Supplementary Figure 5 The expression of TNFRF in mTECs. Quantitative Real-Time PCR analysis of *Cd40*, *Rank*, and *Ltbr* mRNA expression in mTECs (CD45^–^EpCAM^+^UEA-1^+^Ly51^–^) sorted from wild-type and *Sirt6* cKO mice. Data were normalized to *Hprt* mRNA levels. ^∗^*p* < 0.05 (Student’s *t*-test).

Supplementary Figure 6 SPIB expression was up-regulated in *Sirt6* cKO TECs. **(A)** The scatter plot showed the difference of transcription factors between wild-type and Sirt6 deficient mTECs. Genes with significant changes were color-coded in the plot. Red indicated that genes up-regulated in *Sirt6* cKO mTECs, while blue indicated that genes down-regulated in *Sirt6* cKO mTECs. The color of genes was under the criterion of *p* < 0.05. **(B)** The original picture of western blot result for Figure 4H. **(C)** Sorting strategies for Figures 4I,J.

Supplementary Figure 7 *Sirt6* deficient mTECs began to decrease in neonates, but not in E16.5. **(A)** Frequencies and absolute numbers of TECs (CD45^–^EpCAM^+^), cTECs (CD45^–^EpCAM^+^UEA-1^–^Ly51^+^) and mTECs (CD45^–^EpCAM^+^UEA-1^+^Ly51^–^) obtained from E16.5 *Sirt6* cKO mice and littermates. **(B)** Frequencies and absolute numbers of TECs (CD45^–^EpCAM^+^), cTECs (CD45^–^EpCAM^+^UEA-1^–^Ly51^+^), mTECs (CD45^–^EpCAM^+^UEA-1^+^Ly51^–^), CD80^+^ mTECs (CD45^–^EpCAM^+^UEA-1^+^Ly51^–^CD80^+^) obtained from WT and *Sirt6* cKO neonatals. *N* ≥ 4 per group. ^∗^*p* < 0.05 and ^∗∗^*p* < 0.01 (Student’s *t*-test).

Supplementary Figure 8 The development of thymocytes in WT and *Sirt6* cKO mice. **(A)** Gating strategies for Figure 5C. **(B)** FACS plots for Figures 5D,E. **(C)** Absolute numbers of CD4^+^ splenocytes and CD8^+^ splenocytes of wild-type and *Sirt6* cKO mice. **(D)** Gating strategies for Figure 5I.

## Abbreviations

BrdU: 5-bromo-2′-deoxyuridine
cTEC: cortical thymic epithelial cells
DN: double negative
DP: double positive
ETPs: early thymic precursors
GSEA: Gene Set Enrichment Analysis
IKK: I κ B-kinase
LT β R: lymphotoxin β receptor
mTECs: medullary thymic epithelial cells
MHC II: major histocompatibility complex class II
NIK: NF- κ B inducing kinase
nTreg: thymic regulatory T cell
OPG: osteoprotegerin
RANK: receptor activator of NF- κ B
SP: single positive
TECs: thymic epithelial cells
TRAs: tissue restricted antigens
TRAF6: TNF receptor-activated factor 6
TNFRF: TNF receptor family
UEA1: Ulex Europaeus Lectin 1.
