# Supplementary material for: Sirt6 Regulates the Development of Medullary Thymic Epithelial Cells and Contributes to the Establishment of Central Immune Tolerance
Source: Front Cell Dev Biol. 2021 Mar 29;9:655552. doi: 10.3389/fcell.2021.655552 (PMC8044826; doi:10.3389/fcell.2021.655552)

Sirt6 regulates the development of medullary thymic epithelial cells and contributes to the establishment of central immune tolerance

Qian Zhang[1,2,#], Zhanfeng Liang[1,2,#], Jiayu Zhang[1,2,#], Tong Lei[2,#], Xue Dong[1,2], Huiting Su[3], Yifang Chen[1,2], Zhaoqi Zhang[1,2], Liang Tan[4], Yong Zhao[1,2,5]

[1] State Key Laboratory of Membrane Biology, Institute of Zoology, Chinese Academy of Sciences, Beijing, China; [2] University of Chinese Academy of Sciences, Beijing, China; [3] Central Laboratory of Peking university people's hospital, Beijing, China; [4] Center of Organ Transplantation, Second Xiangya Hospital of Central South University, Changsha, China; [5] Institute for Stem Cell and Regeneration, Chinese Academy of Sciences, Beijing, China.

Supplementary Figure 1

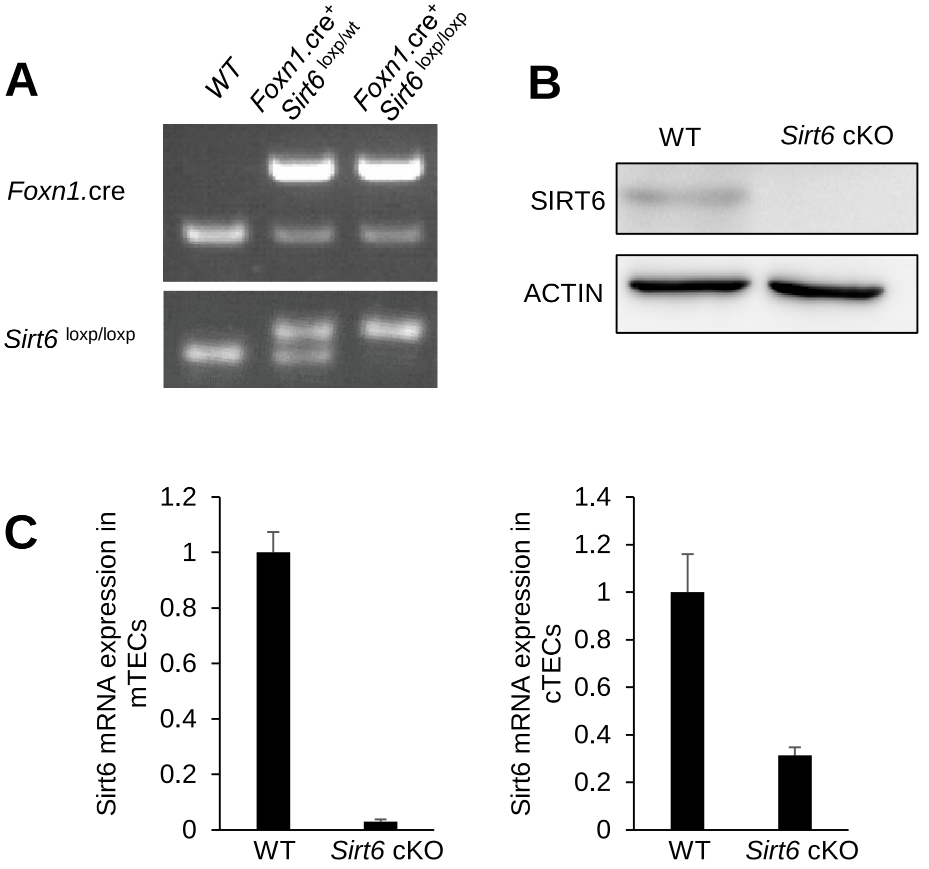

Supplementary Figure 2

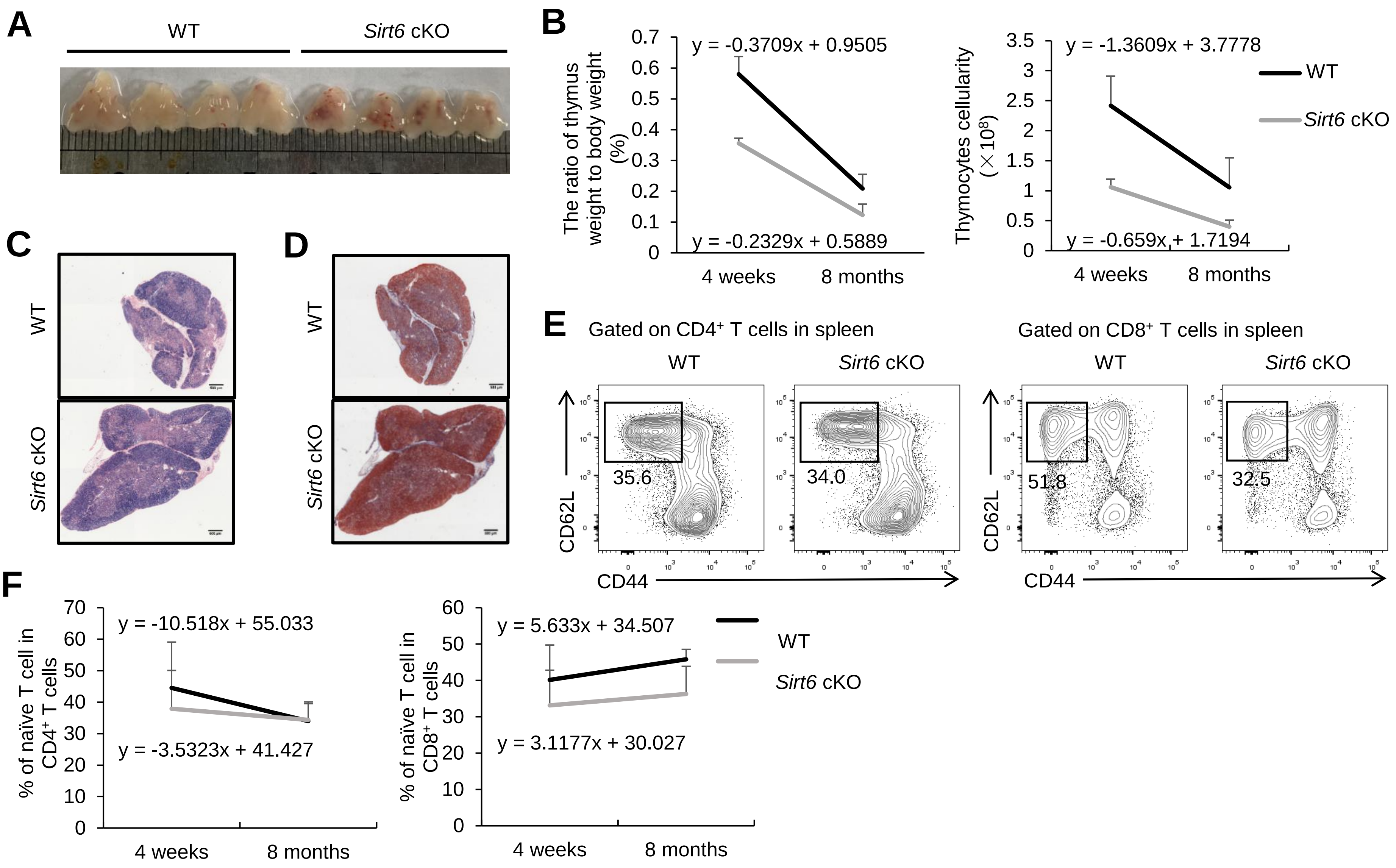

Supplementary Figure 3

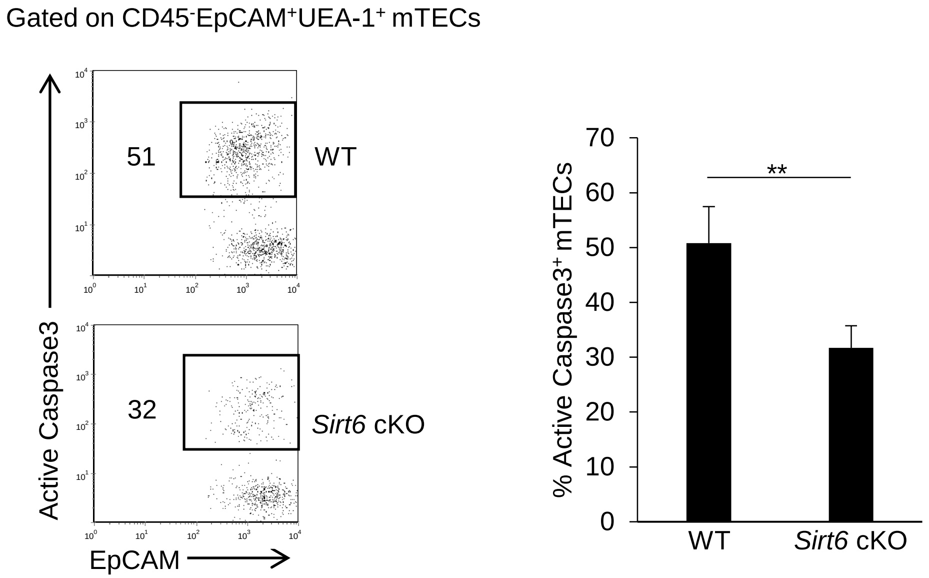

Supplementary Figure 4

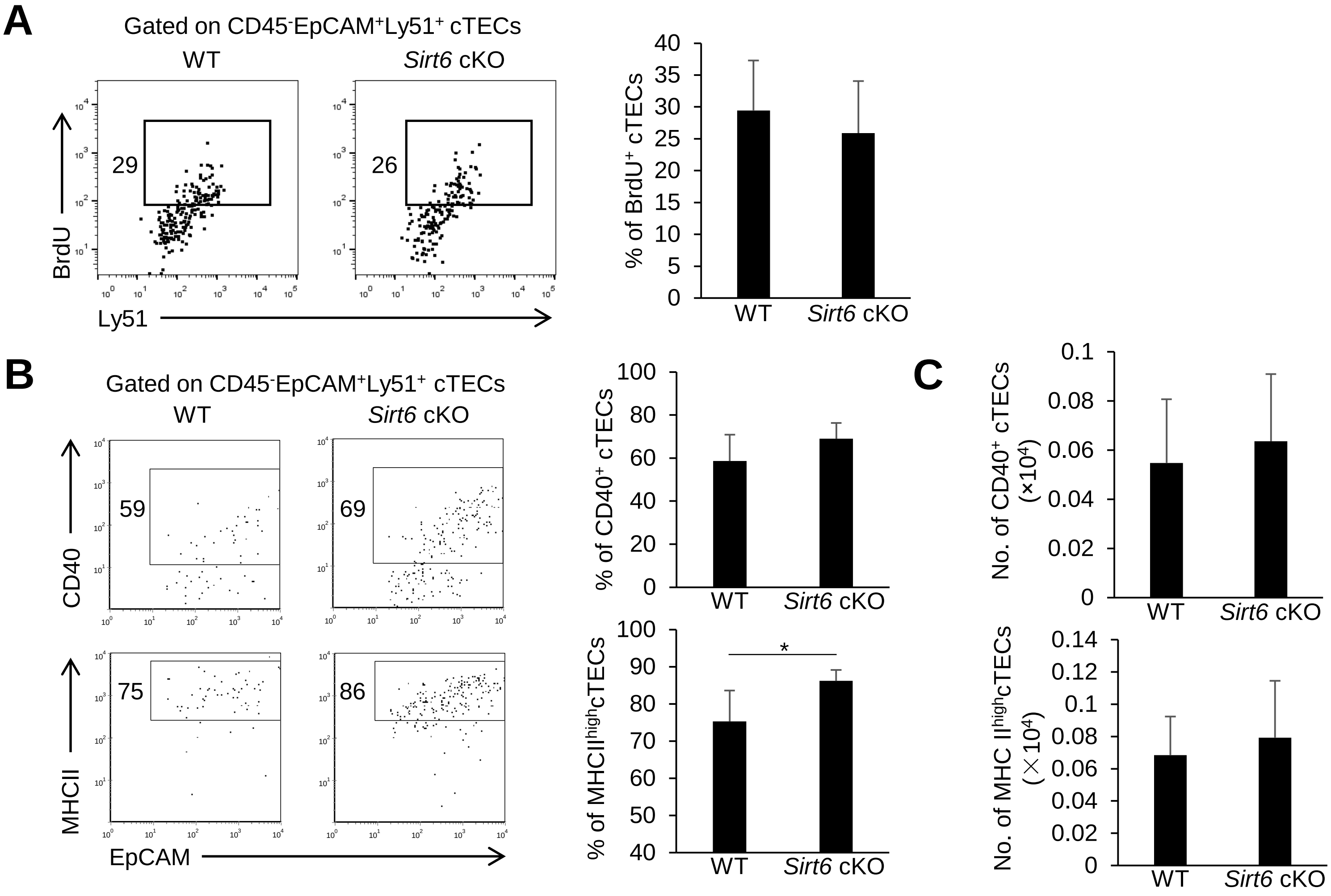

Supplementary Figure 5

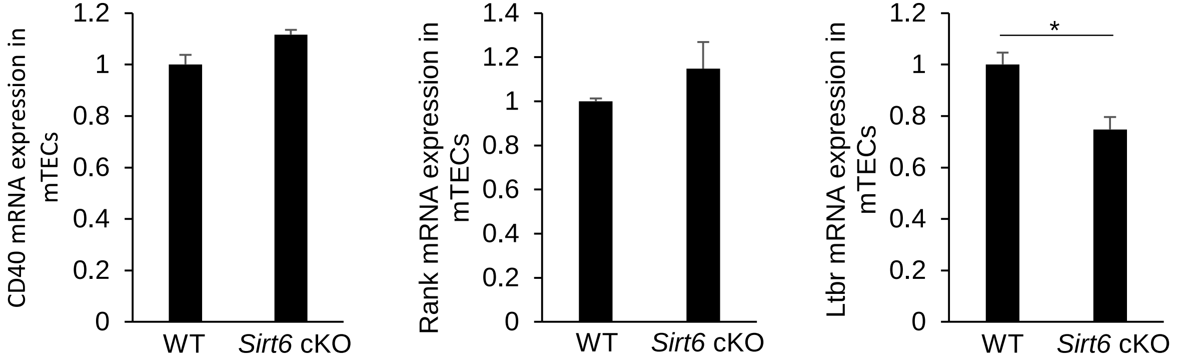

Supplementary Figure 6

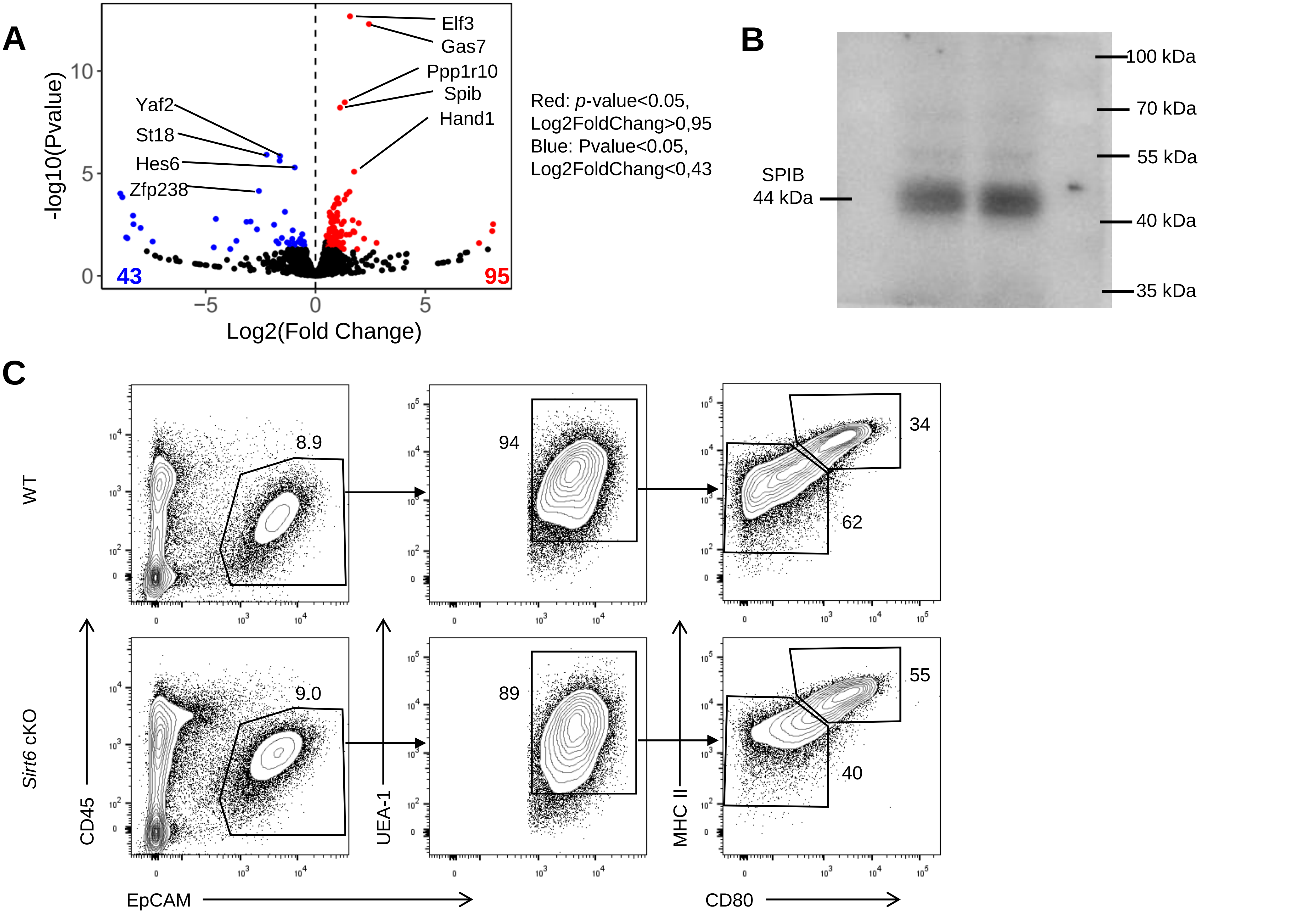

Supplementary Figure 7

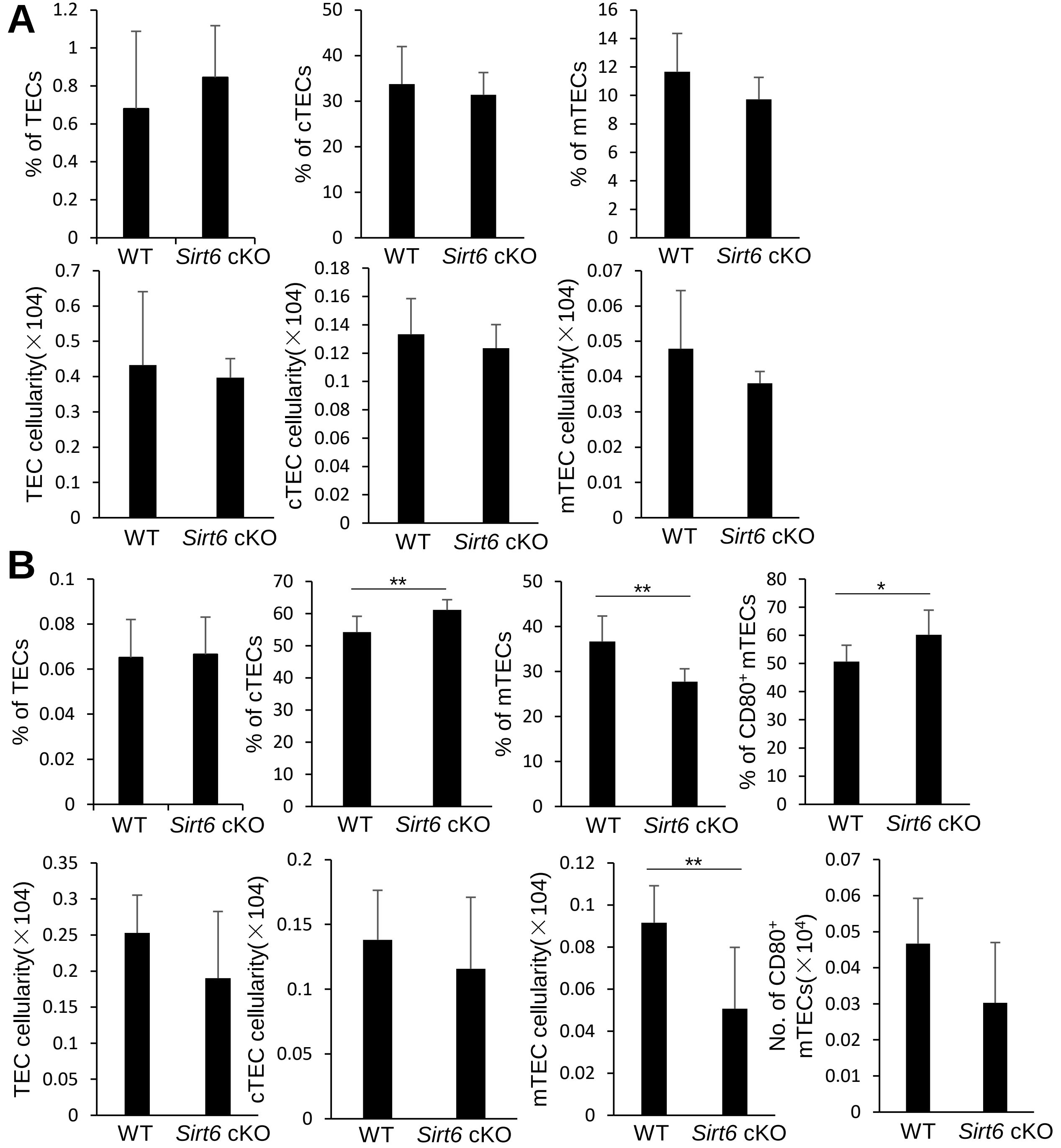

Supplementary Figure 8

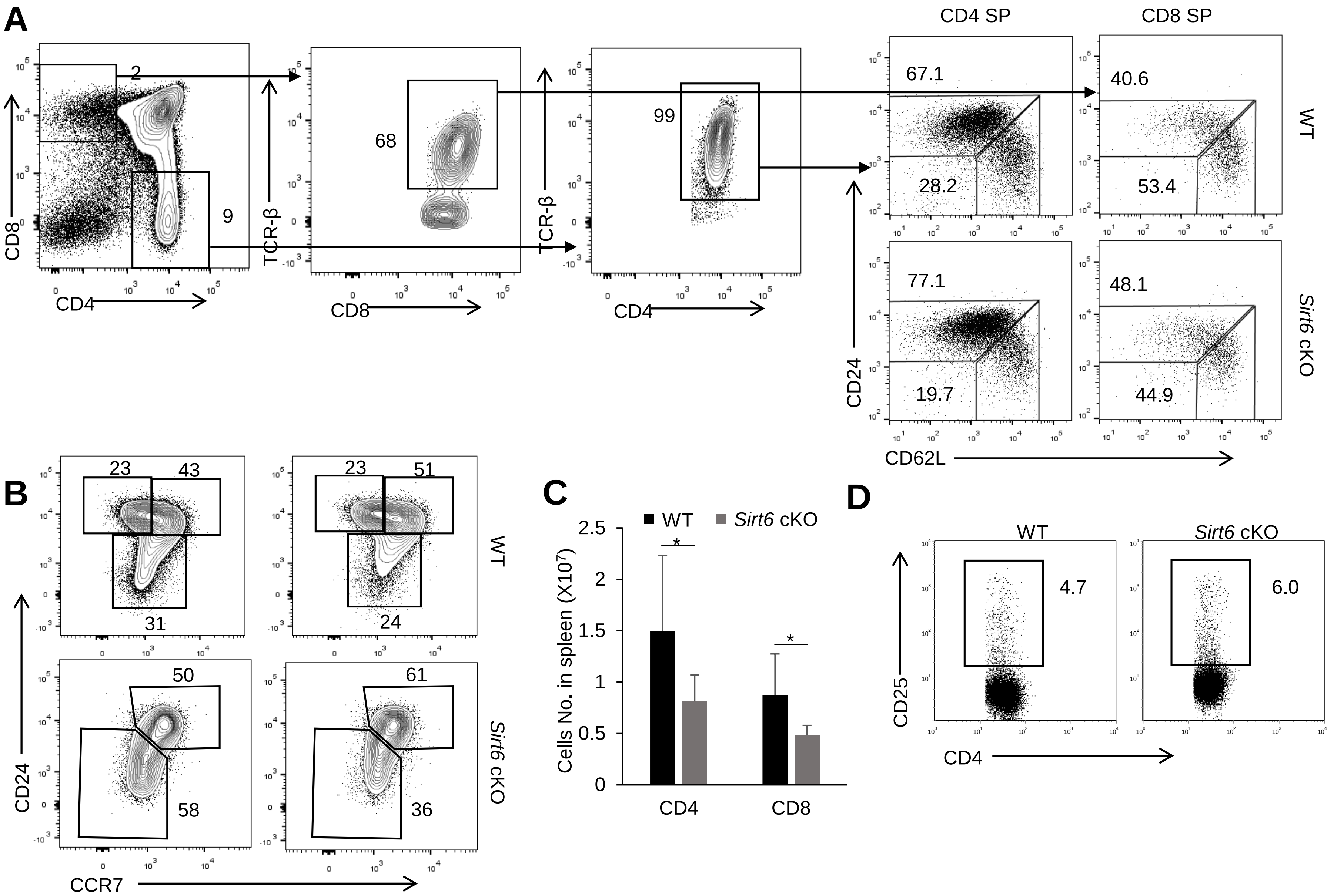

Supplement: Supplementary Figure 1 — SIRT6 was successfully knockout in TECs. (A) Identification method of WT (Foxn1.cre–) and Sirt6 cKO (Foxn1.cre+, Sirt6loxp/loxp) mice. (B) Western blot result for Foxn1.Cre-mediated recombination efficacy of Sirt6loxp/loxp between wild-type TECs and Sirt6 cKO TECs cultured in vitro. (C) Quantitative Real-Time PCR analysis of Sirt6 mRNA expression in mTEC (CD45–EpCAM+UEA-1+Ly51–) and cTEC (CD45–EpCAM+UEA-1–Ly51+) sorted from wild-type and Sirt6 cKO mice. Data were normalized to Hprt mRNA levels. [file Data_Sheet_1.pdf]
